# Endoplasmic reticulum involvement in yeast cell death

**DOI:** 10.3389/fonc.2012.00087

**Published:** 2012-08-02

**Authors:** Nicanor Austriaco, O. P.

**Affiliations:** Department of Biology, Providence College,Providence, RI, USA

**Keywords:** BXI1, endoplasmic reticulum, ER stress, IRE1, UPR, yeast cell death

## Abstract

Yeast cells undergo programed cell death (PCD) with characteristic markers associated with apoptosis in mammalian cells including chromatin breakage, nuclear fragmentation, reactive oxygen species generation, and metacaspase activation. Though significant research has focused on mitochondrial involvement in this phenomenon, more recent work with both *Saccharomyces cerevisiae* and *Schizosaccharomyces pombe* has also implicated the endoplasmic reticulum (ER) in yeast PCD. This minireview provides an overview of ER stress-associated cell death (ER-SAD) in yeast. It begins with a description of ER structure and function in yeast before moving to a discussion of ER-SAD in both mammalian and yeast cells. Three examples of yeast cell death associated with the ER will be highlighted here including inositol starvation, lipid toxicity, and the inhibition of *N*-glycosylation. It closes by suggesting ways to further examine the involvement of the ER in yeast cell death.

## INTRODUCTION

In recent years, it has become increasingly clear that yeast cells undergo programed cell death (PCD) in response to a variety of intrinsic and extrinsic stimuli, with characteristic markers associated with apoptosis in mammalian cells (Carmona-Gutierrez et al., 2010). Significantly, yeast orthologs of crucial metazoan apoptotic proteins, which include the metacaspase, Yca1p (Madeo et al., 2002), the yeast AIF1 homolog, Aif1p (Wissing et al., 2004), and the endonuclease G homolog, Nuc1p (Buttner et al., 2007), have been identified and linked to yeast cell death suggesting that a core machinery driving PCD is conserved in unicellular eukaryotes (Madeo et al., 2009).

Though significant research has focused on mitochondrial involvement in yeast PCD (Braun and Westermann, 2011), recent work with both *Saccharomyces cerevisiae* and *Schizosaccharomyces pombe* has implicated the endoplasmic reticulum (ER) in yeast PCD. This minireview provides an overview of ER stress-associated cell death (ER-SAD) in yeast. It begins with a description of ER structure and function in yeast before moving to a discussion of ER-SAD in both mammalian and yeast cells. It closes by suggesting ways to further examine the involvement of the ER in yeast cell death.

## ENDOPLASMIC RETICULUM STRUCTURE IN YEAST

The ER is the largest membrane-bound organelle in the eukaryotic cell (Friedman and Voeltz, 2011; Hu et al., 2011). It consists of the nuclear envelope and the peripheral ER, a single network of interconnected sheets and tubules, which in yeast is located close to the plasma membrane where it is referred to as the cortical ER. A recent study that imaged the budding yeast ER by transmission electron microscopy and dual-axis electron tomography revealed that it can divided into three structurally distinct major domains: the plasma membrane-associated ER (pmaER), the central cisternal ER (cecER), and the tubular ER (tubER; West et al., 2011).

Molecular components involved in regulating ER structure have recently been identified. Regions of high membrane curvature including the edges of the sheets and the tubules are stabilized by interactions between members of the reticulon family (Rtn1p and Rtn2p in budding yeast) and members of the DP1/Yop1p family of proteins (Yop1p in budding yeast; Voeltz et al., 2006; Shibata et al., 2010). It is thought that Rtn1p, Rtn2p, and Yop1p, like their mammalian counterparts, stabilize the high curvature of ER tubules by using their double hairpin structure to form wedges and arc-like scaffolds that mold the lipid bilayer into tubules (Shibata et al., 2009, 2010).

Once ER tubules are shaped and formed, they need to be connected to the ER network via homotypic fusion between two identical but apposing membranes. In yeast, homotypic fusion appears to be mediated by the Sey1p protein, the ortholog of the mammalian atlastins, a class of GTPases that belong to the dynamin family (Hu et al., 2009; Anwar et al., 2012). Given Sey1p’s structure, the data suggest that the fusion reaction could be mediated by conformational changes in the Sey1p GTPase domains that pull the apposing membranes together forcing them to fuse (Orso et al., 2009; Anwar et al., 2012).

## ENDOPLASMIC RETICULUM FUNCTION IN YEAST

In all eukaryotic cells, the ER performs a variety of functions including protein translocation and folding, lipid synthesis, and calcium homeostasis. In yeast cells, protein transport into the ER can occur via either the signal recognition particle (SRP)-dependent (cotranslational translocation) or the Hsp70p-dependent (post-translational translocation) pathway (Zimmermann et al., 2011). Membrane insertion and completion of translocation involve the heterotrimeric Sec61p complex and the ER-lumenal chaperone, Kar2p (or BiP). Kar2p appears to facilitate post-translational insertion of the polypeptide into the Sec61p complex and its translocation via a ratcheting mechanism (Lyman and Schekman, 1996). Once ER client proteins have been translocated, they can be folded, modified, and packaged into ER-to-Golgi COPII transport vesicles that form at ER exit sites (ERES; Duden, 2003; Watanabe and Riezman, 2004; Watson and Stephens, 2005). In yeast, ER sheets have a markedly higher ribosome density than tubules suggesting that sheets may be better suited for ribosome binding and protein translocation (West et al., 2011).

Next, the ER serves as the main site for synthesis of three major classes of membrane lipids: sphingolipids, phospholipids, and sterols (Carman and Henry, 2007; Henry et al., 2012). For example, in yeast, most of the biosynthetic steps for triacylglycerols occur in the ER: Gat1p and Gat2p, which are the major glycerol-3-phosphate acyltransferases (GPATs) that catalyze the first step in the synthesis of almost all membrane phospholipids and neutral glycerolipids, localize to both the perinuclear and the cortical ER (Bratschi et al., 2009). Additionally, the two ER membrane proteins, Orm1p and Orm2p, have been shown to be involved in sphingolipid synthesis and phospholipid homeostasis (Han et al., 2010). These are only a few of numerous ER-localized gene products known to be involved in lipid biosynthesis in yeast.

Finally, the creation of a specifically targeted version of the Ca^2+^-sensitive photoprotein, aequorin, to the lumen of the yeast ER revealed that this organelle is involved in calcium storage and homeostasis with a steady-state concentration of 10 μM free Ca^2+^ in wild-type yeast cells (Strayle et al., 1999). It is also known that two P-type ATPases are involved in regulating the levels of Ca^2+^ in the ER including the yeast high-affinity Ca^2+^/Mn^2+^ P-type ATPase, Pmr1p, which pumps cytosolic Ca^2+^ into the ER and the Golgi (Sorin et al., 1997; Strayle et al., 1999), and the ER-localized P-type ATPase, Cod1p/Spf1p, which appears to work with Pmr1p to maintain ER function and homeostasis (Vashist et al., 2002). Significantly, Ca^2+^ levels in the ER have been implicated in retention of resident luminal proteins, in export of secretory proteins, in protein folding and degradation, and in the association of the ER chaperone Kar2p with misfolded proteins (Durr et al., 1998).

## ER STRESS-ASSOCIATED DEATH IN MAMMALIAN CELLS

In mammalian cells, a diverse range of factors can disrupt ER function and lead to ER stress, which if left unchecked can trigger ER-SAD. These include increases in ER-lumenal protein levels that exceed the capacity of ER-resident chaperones, exposure to long-chain saturated fatty acids (SFA), alterations in calcium levels in the ER lumen, and disturbances to the ER redox balance (Ron and Walter, 2007; Parmar and Schroder, 2012). In animal cells, ER stress is sensed by three upstream signaling pathways driven by three effector proteins, IRE1, ATF6, and PERK, which are collectively called the unfolded protein response (UPR; Wang et al., 1998; Harding et al., 1999; Urano et al., 2000; Walter and Ron, 2011). Activation of the UPR can alleviate ER stress by synthesizing novel components of the protein folding machinery and by expanding the ER itself (Cox et al., 1997; Schuck et al., 2009). This activated UPR can be divided into three phases: the adaptive, the alarm, and the apoptotic phase. The adaptive phase begins with an immediate and fast response that decreases protein influx into the ER followed by a slower transcriptional response involving downstream transcription factors that upregulate genes encoding ER-resident chaperones, components of the ER-associated protein degradation (ERAD) machinery, and regulators of ER size (Trusina et al., 2008). In many scenarios, the adaptive phase can restore equilibrium between protein load and chaperone capacity within the ER, dampening the UPR. However, in cases of chronic or unresolved ER stress, the UPR continues to a second alarm phase that involves several signal transduction events that move the cell from a pro-survival to a pro-apoptotic state (Tabas and Ron, 2011; Woehlbier and Hetz, 2011). This ends in the final apoptosis phase involving the transcriptional and post-translational activation of the BH3-only proteins and other BCL2 protein family members that trigger the canonical mitochondrial cell death pathway. Additionally, the BCL2 proteins have been implicated in linking ER Ca^2+^ homeostasis and apoptosis (Oakes et al., 2003; Bassik et al., 2004).

Mechanistically, in mammalian cells, the ER-SAD program is mediated largely by the UPR signaling molecules and endonuclease IRE1α, which can transmit both pro-survival and pro-death signals. Indeed, in my view, ER-SAD can be defined as the cell death process that involves IRE1 function. In light of this, it is significant that there are pro- and anti-apoptotic effectors assembled around IRE1α that are able to control the amplitude and duration of IRE1α signaling (Woehlbier and Hetz, 2011). For example, IRE1α signaling can be enhanced at the ER membrane by the formation of a complex between the cytosolic domains of IRE1α and the BAX–BAK complex, two pro-apoptotic members of the BCL2 family of proteins (Scorrano et al., 2003; Zong et al., 2003). In contrast, IRE1α signaling can be attenuated by the binding of the ER-localized anti-apoptotic protein, BAX inhibitor-1 (BI-1; Lisbona et al., 2009; Bailly-Maitre et al., 2010; Castillo et al., 2011). Late-phase UPR signaling of IRE1α can lead to changes in the expression and activity of BCL2 protein family members and therefore to the activation of apoptosis.

Finally, we should note two emerging areas of inquiry involving the role of the ER in cell death. First, several studies in mammalian cells have begun exploring the links between ER and mitochondrial function during PCD. Both organelles form interconnected membrane networks that can influence various cellular processes including cell death (Csordas et al., 2006). More recently, Cardenas and colleagues have shown that Ca^2+^ transport between the ER and the mitochondria is regulated by the inositol triphosphate receptor, IP_3_R, to modulate mitochondrial bioenergetics (Cardenas et al., 2010), a process involving BI-1, an ER-resident protein known to be involved in autophagy and apoptosis (Sano et al., 2012). This may explain the earlier observation that mitochondria preferentially accumulate Ca^2+^ in regions called mitochondria-associated microdomains (MAMs) where the ER and mitochondria are found in close proximity (Rizzuto et al., 1998). Next, there have been a few published reports that describe the fragmentation of the ER during ER stress and ER-SAD in mammalian cells (Brough et al., 2005; Kucharz et al., 2011a,b; Howarth et al., 2012). Though there is evidence that the loss of the GTPase atlastin 1 can cause ER fragmentation in *Drosophila* (Orso et al., 2009), the mechanism behind the ER fragmentation associated with ER stress in the mammalian system is still not known.

## ER STRESS-ASSOCIATED DEATH IN YEAST CELLS

As they do in mammalian cells, a range of intrinsic and extrinsic triggers can disrupt ER function and lead to ER stress and to ER-SAD in yeast (**Table 1**). Notably, however, in yeast cells, ER stress is only sensed by a single signaling pathway driven by Ire1p, which is the most ancient of the three parallel UPR pathways found in the metazoan (Sidrauski and Walter, 1997). Three examples of yeast cell death associated with the ER will be highlighted here, including inositol starvation, lipid toxicity, and the inhibition of *N*-glycosylation.

**Table 1:** ER-Associated Cell Death in Mammalian and Yeast Cells.

ER function	Organism	Triggers of cell death (select list)	ER proteins involved
Protein translocation and folding	Mammalian	Protein aggregation	IRE1α
		Ischemia reperfusion	ATF6
		Beta-mercaptoethanol (BME)	PERK
		Tunicamycin	BI
		Dithiothreitol (DTT)	Grp78/BiP
	Yeast	Heat shock	Ire1p
		Beta-mercaptoethanol (BME)	Bxi1p
		Tunicamycin	Kar2p/BiP
		Dithiothreitol (DTT)	
Lipid synthesis	Mammalian	Elevated free fatty acids (FFA)	IRE1α
			ATF6
			PERK
	Yeast	Elevated saturated fatty acids	Ire1p
		(SFA) inositol starvation	Cnx1p
Calcium dynamics	Mammalian	Thapsigargin	PERK
		Calcium overload	SERCA
			Grp78/BiP
	Yeast	Calcium starvation	Pmr1p
		Calcium chelators	

First, Guerin et al. (2009) reported that the fission yeast, *S. pombe*, undergoes cell death with apoptotic-like features when it is deprived of inositol, a precursor of numerous phospholipids and signaling molecules. Deleting either *pca1*^+^, the gene for the only caspase-like protein in *S. pombe*, or *ire1*^+^, the gene for the only IRE1 homolog, enhanced cell survival in media lacking inositol, suggesting that both genes are involved in the cell death pathway. Interestingly, the ER transmembrane chaperone, calnexin, encoded by the *cnx1*^+^ gene, has also been implicated in inositol-starvation-induced ER-SAD since overexpressing different portions of the Cnx1p protein can alter the number of dying cells in inositol-starved conditions as measured by several assays (Guerin et al., 2009).

Next, feeding the budding yeast, *S. cerevisiae*, with extracellular SFA like palmitate (C16:0) triggers ER stress and leads to growth arrest and death (Pineau et al., 2009). Addition of palmitate to the cell culture also alters ER morphology with swelling of the organelle, detachment of the pmaER from the plasma membrane, and in certain cells, the replacement of pmaER by electron-lucent clefts extending throughout the cytoplasm that are visible in the electron microscope. Both the induction of the UPR and the cell death associated with lipid toxicity were abrogated with the addition of the molecular chaperone 4-phenyl butyrate, suggesting that lipid-induced ER stress overburdens the folding machinery in the ER. Notably, lipid toxicity is known to induce apoptosis in mammalian cells (Kharroubi et al., 2004; Diakogiannaki et al., 2008). One paper reports that palmitate induces apoptosis in pancreatic beta cells by activating the IRE1α, PERK, and ATF6 pathways (Cunha et al., 2008).

Finally, Hauptmann et al. (2006) reported that preventing the *N*-glycosylation of yeast proteins, either by mutating critical subunits of the oligosaccharyltransferase (OST) complex in the ER lumen, or by treating the cells with tunicamycin, a drug known to block the ER enzyme UDP-*N*-acetylglucosamine-1-*P* transferase (Alg7p) that is necessary for *N*-glycosylation, induced an apoptotic-like death in wild-type *S. cerevisiae* cells. The dying cells contained condensed nuclei, fragmented DNA, and externalized phosphatidylserine. Defects in *N*-glycosylation also led both to the appearance of a caspase-like activity that did not require functional yeast metacaspase, Yca1p, and to the production of reactive oxygen species (ROS) that could be diminished by heterologous expression of the human anti-apoptotic protein, Bcl-2. Two years later, the same team reported that the Golgi-localized Kex1p protease is involved in the apoptotic-like cell death linked to defects in *N*-glycosylation (Hauptmann and Lehle, 2008). Deletion of *KEX1* diminished the appearance of the caspase-like activity and decreased ROS accumulation in cells cultured in tunicamycin. Notably, the cell death described in these experiments was blocked by the addition of osmotic stabilizers to the culture media.

Strikingly, these studies disagreed with previous findings that had shown that tunicamycin does not induce cell death in wild-type cells unless the calcium-dependent phosphatase, calcineurin, had previously been inactivated (Bonilla et al., 2002; Bonilla and Cunningham, 2003). To resolve this disagreement, Dudgeon et al. (2008) used improved staining methods using both propidium iodide (PI) and FITC-VAD-FMK together instead of FITC-VAD-FMK alone, to analyze the cell death associated with growth in media containing tunicamycin. Their data showed that tunicamycin can induce cell death, but only in cells grown in low osmolyte yeast–peptone–dextrose (YPD) media, and not in cells grown in synthetic media. They also demonstrated that this dying process is not apoptotic in nature. The dying cells lacked two critical hallmarks of apoptosis – both chromatin fragmentation and phosphatidylserine externalization – suggesting that there may have been methodological problems in past efforts to characterize the cell death induced by tunicamycin in *S. cerevisiae*.

Instead, tunicamycin appeared to trigger two different forms of death in wild-type budding yeast cells, one that is partially dependent on a functional electron transport chain (ETC) and another that is independent of ETC function. Active calcineurin signaling could prevent the former, which is why it has been called calcineurin-less death, but not the latter form of cell death. Significantly, calcineurin-less death in response to tunicamycin did not involve any of the apoptosis-associated factors, Yca1p, Nuc1p, Nma111p, or Ste20p, strengthening the claim that this death is not apoptotic in nature. Rather, the dying process was regulated by Cmk2p, one of the two Ca^2+^/calmodulin-dependent protein kinases, and by Hsp90p, one of the major heat shock chaperones in budding yeast. Finally, a very recent study from the same research team has concluded that tunicamycin leads to cell death by permeabilizing the vacuolar membranes of yeast cells and that this cell death program involves the V-ATPase that acidifies the vacuole (Kim et al., 2012). This tunicamycin-induced cell death involving vacuole membrane permeabilization could be blocked by calmodulin.

In light of the cumulative evidence, in my view, it is clear that tunicamycin leads to cell death, but that this dying process is not apoptotic in nature as had been previously reported. One possible reason for the discrepancy in the literature is the earlier study’s failure to distinguish living apoptotic cells from dead necrotic cells, which are known to stain non-specifically with FITC-VAD-FMK (Wysocki and Kron, 2004; Hauptmann et al., 2006). Moreover, the observation reported in the earlier paper that the addition of an osmotic stabilizer like sorbitol is able to block some of the *N*-glycosylation-linked cell death suggests that this cell death may be lytic rather than apoptotic in nature (Hauptmann and Lehle, 2008). This is not surprising since protein *N*-glycosylation is crucial for cell wall assembly (Lesage and Bussey, 2006; Levin, 2011). Defects in this biochemical pathway would be expected to weaken the cell’s ability to maintain its structural integrity.

Finally, can this tunicamycin-induced calcineurin-less cell death in *S. cerevisiae* properly be called ER stress-associated cell death? It is not clear. As I noted above, in mammalian cells, ER-SAD has been linked to the activation of the UPR and IRE1 function. Though tunicamycin does induce the UPR in budding yeast, it is also apparent that Ire1p is not required for either the pro-death activity of tunicamycin in calcineurin-deficient cells or the anti-death activity of calcineurin in these cells (Bonilla et al., 2002). This is in stark contrast to the single published report suggesting that tunicamycin induces an apoptotic-like cell death in *S. pombe* that appears to partially require the fission yeast Ire1p (Guerin et al., 2008).

## FUTURE DIRECTIONS

The study of the ER’s role in yeast cell death is in its infancy. It would be interesting to determine if other physiological triggers of yeast cell death like acetic acid or ethanol – triggers not directly linked to ER function – involve the ER. Recently, my laboratory has begun characterizing the yeast BI protein, Bxi1p, an ER-localized protein that links the UPR to PCD in *S. cerevisiae* (Cebulski et al., 2011).Cells lacking *BXI1* are not only more sensitive both to ethanol-induced and to glucose-induced PCD, but also have a diminished UPR. Studies linking Bxi1p and Ire1p function in yeast are ongoing in my lab. Finally, it will be important to check if PCD alters the yeast’s ER structure, and conversely, if mutants that modify ER structure alter the dynamics of yeast cell death. These investigations should uncover the mechanistic links between ER structure, ER function, and cell death in yeast.

## Conflict of Interest Statement

The author declares that the research was conducted in the absence of any commercial or financial relationships that could be construed as a potential conflict of interest.

## References

[B1] Anwar K. Klemm R. W. Condon A. Severin K. N. Zhang M. Ghirlando R. Hu J. Rapoport T. A. Prinz W. A. (2012). The dynamin-like GTPase Sey1p mediates homotypic ER fusion in *S. cerevisiae*. *J. Cell Biol.* 197 209–217 22508509 10.1083/jcb.201111115 PMC3328390

[B2] Bailly-Maitre B. Belgardt B. F. Jordan S. D. Coornaert B. von Freyend M. J. Kleinridders A. Mauer J. Cuddy M. Kress C. L. Willmes D. Essig M. Hampel B. Protzer U. Reed J. C. Bruning J. C. (2010). Hepatic Bax inhibitor-1 inhibits IRE1alpha and protects from obesity-associated insulin resistance and glucose intolerance. *J. Biol. Chem.* 285 6198–6207 19996103 10.1074/jbc.M109.056648 PMC2825415

[B3] Bassik M. C. Scorrano L. Oakes S. A. Pozzan T. Korsmeyer S. J. (2004). Phosphorylation of BCL-2 regulates ER Ca^2+^ homeostasis and apoptosis. *EMBO J.* 23 1207–1216 15010700 10.1038/sj.emboj.7600104 PMC380968

[B4] Bonilla M. Cunningham K. W. (2003). Mitogen-activated protein kinase stimulation of Ca^2+^ signaling is required for survival of endoplasmic reticulum stress in yeast. *Mol. Biol. Cell* 14 4296–4305 14517337 10.1091/mbc.E03-02-0113 PMC207020

[B5] Bonilla M. Nastase K. K. Cunningham K. W. (2002). Essential role of calcineurin in response to endoplasmic reticulum stress. *EMBO J.* 21 2343–2353 12006487 10.1093/emboj/21.10.2343 PMC126012

[B6] Bratschi M. W. Burrowes D. P. Kulaga A. Cheung J. F. Alvarez A. L. Kearley J. Zaremberg V. (2009). Glycerol-3-phosphate acyltransferases gat1p and gat2p are microsomal phosphoproteins with differential contributions to polarized cell growth. *Eukaryot. Cell* 8 1184–1196 19525420 10.1128/EC.00085-09 PMC2725570

[B7] Braun R. J. Westermann B. (2011). Mitochondrial dynamics in yeast cell death and aging. *Biochem. Soc. Trans*. 39 1520–1526 21936845 10.1042/BST0391520

[B8] Brough D. Sim Y. Thorn P. Irvine R. F. (2005). The structural integrity of the endoplasmic reticulum, and its possible regulation by inositol 1,3,4,5-tetrakisphosphate. *Cell Calcium* 38 153–159 16023721 10.1016/j.ceca.2005.05.002

[B9] Buttner S. Eisenberg T. Carmona-Gutierrez D. Ruli D. Knauer H. Ruckenstuhl C. Sigrist C. Wissing S. Kollroser M. Frohlich K. U. Sigrist S. Madeo F. (2007). Endonuclease G regulates budding yeast life and death. *Mol. Cell* 25 233–246 17244531 10.1016/j.molcel.2006.12.021

[B10] Cardenas C. Miller R. A. Smith I. Bui T. Molgo J. Muller M. Vais H. Cheung K. H. Yang J. Parker I. Thompson C. B. Birnbaum M. J. Hallows K. R. Foskett J. K. (2010). Essential regulation of cell bioenergetics by constitutive InsP3 receptor Ca^2+^ transfer to mitochondria. *Cell* 142 270–283 20655468 10.1016/j.cell.2010.06.007 PMC2911450

[B11] Carman G. M. Henry S. A. (2007). Special issue: regulation of lipid metabolism in yeast. *Biochim. Biophys. Acta* 1771 239–240 18311317 10.1016/j.bbalip.2006.11.001 PMC1868683

[B12] Carmona-Gutierrez D. Eisenberg T. Buttner S. Meisinger C. Kroemer G. Madeo F. (2010). Apoptosis in yeast: triggers, pathways, subroutines. *Cell Death Differ.* 17 763–773 20075938 10.1038/cdd.2009.219

[B13] Castillo K. Rojas-Rivera D. Lisbona F. Caballero B. Nassif M. Court F. A. Schuck S. Ibar C. Walter P. Sierralta J. Glavic A. Hetz C. (2011). BAX inhibitor-1 regulates autophagy by controlling the IRE1alpha branch of the unfolded protein response. *EMBO J.* 30 4465–4478 21926971 10.1038/emboj.2011.318 PMC3230375

[B14] Cebulski J. Malouin J. Pinches N. Cascio V. Austriaco N. (2011). Yeast Bax inhibitor, Bxi1p, is an ER-localized protein that links the unfolded protein response and programmed cell death in *Saccharomyces cerevisiae*. *PLoS ONE* 6 e20882 10.1371/journal.pone.0020882 PMC3108976 21673967

[B15] Cox J. S. Chapman R. E. Walter P. (1997). The unfolded protein response coordinates the production of endoplasmic reticulum protein and endoplasmic reticulum membrane. *Mol. Biol. Cell* 8 1805–1814 9307975 10.1091/mbc.8.9.1805 PMC305738

[B16] Csordas G. Renken C. Varnai P. Walter L. Weaver D. Buttle K. F. Balla T. Mannella C. A. Hajnoczky G. (2006). Structural and functional features and significance of the physical linkage between ER and mitochondria. *J. Cell Biol.* 174 915–921 16982799 10.1083/jcb.200604016 PMC2064383

[B17] Cunha D. A. Hekerman P. Ladriere L. Bazarra-Castro A. Ortis F. Wakeham M. C. Moore F. Rasschaert J. Cardozo A. K. Bellomo E. Overbergh L. Mathieu C. Lupi R. Hai T. Herchuelz A. Marchetti P. Rutter G. A. Eizirik D. L. Cnop M. (2008). Initiation and execution of lipotoxic ER stress in pancreatic beta-cells. *J. Cell Sci.* 121(Pt 14) 2308–2318 18559892 10.1242/jcs.026062 PMC3675788

[B18] Diakogiannaki E. Welters H. J. Morgan N. G. (2008). Differential regulation of the endoplasmic reticulum stress response in pancreatic beta-cells exposed to long-chain saturated and monounsaturated fatty acids. *J. Endocrinol.* 197 553–563 18492819 10.1677/JOE-08-0041

[B19] Duden R. (2003). ER-to-Golgi transport: COP I and COP II function (review). *Mol. Membr. Biol.* 20 197–207 12893528 10.1080/0968768031000122548

[B20] Dudgeon D. D. Zhang N. Ositelu O. O. Kim H. Cunningham K. W. (2008). Nonapoptotic death of *Saccharomyces cerevisiae* cells that is stimulated by Hsp90 and inhibited by calcineurin and Cmk2 in response to endoplasmic reticulum stresses. *Eukaryot. Cell* 7 2037–2051 18806210 10.1128/EC.00291-08 PMC2593186

[B21] Durr G. Strayle J. Plemper R. Elbs S. Klee S. K. Catty P. Wolf D. H. Rudolph H. K. (1998). The medial-Golgi ion pump Pmr1 supplies the yeast secretory pathway with Ca^2+^ and Mn^2+^ required for glycosylation, sorting, and endoplasmic reticulum-associated protein degradation. *Mol. Biol. Cell* 9 1149–1162 9571246 10.1091/mbc.9.5.1149 PMC25337

[B22] Friedman J. R. Voeltz G. K. (2011). The ER in 3D: a multifunctional dynamic membrane network. *Trends Cell Biol.* 21 709–717 21900009 10.1016/j.tcb.2011.07.004 PMC3221873

[B23] Guerin R. Arseneault G. Dumont S. Rokeach L. A. (2008). Calnexin is involved in apoptosis induced by endoplasmic reticulum stress in the fission yeast. *Mol. Biol. Cell* 19 4404–4420 18701708 10.1091/mbc.E08-02-0188 PMC2555920

[B24] Guerin R. Beauregard P. B. Leroux A. Rokeach L. A. (2009). Calnexin regulates apoptosis induced by inositol starvation in fission yeast. *PLoS ONE* 4 e6244 10.1371/journal.pone.0006244 PMC2705804 19606215

[B25] Han S. Lone M. A. Schneiter R. Chang A. (2010). Orm1 and Orm2 are conserved endoplasmic reticulum membrane proteins regulating lipid homeostasis and protein quality control. *Proc. Natl. Acad. Sci. U.S.A.* 107 5851–5856 20212121 10.1073/pnas.0911617107 PMC2851911

[B26] Harding H. P. Zhang Y. Ron D. (1999). Protein translation and folding are coupled by an endoplasmic-reticulum-resident kinase. *Nature* 397 271–274 9930704 10.1038/16729

[B27] Hauptmann P. Lehle L. (2008). Kex1 protease is involved in yeast cell death induced by defective N-glycosylation, acetic acid, and chronological aging. *J. Biol. Chem.* 283 19151–19163 18474590 10.1074/jbc.M801303200

[B28] Hauptmann P. Riel C. Kunz-Schughart L. A. Frohlich K. U. Madeo F. Lehle L. (2006). Defects in N-glycosylation induce apoptosis in yeast. *Mol. Microbiol.* 59 765–778 16420350 10.1111/j.1365-2958.2005.04981.x

[B29] Henry S. A. Kohlwein S. D. Carman G. M. (2012). Metabolism and regulation of glycerolipids in the yeast *Saccharomyces cerevisiae*. *Genetics* 190 317–349 22345606 10.1534/genetics.111.130286 PMC3276621

[B30] Howarth D. L. Vacaru A. M. Tsedensodnom O. Mormone E. Nieto N. Costantini L. M. Snapp E. L. Sadler K. C. (2012). Alcohol disrupts endoplasmic reticulum function and protein secretion in hepatocytes. *Alcohol. Clin. Exp. Res.* 36 14–23 21790674 10.1111/j.1530-0277.2011.01602.x PMC3204333

[B31] Hu J. Prinz W. A. Rapoport T. A. (2011). Weaving the web of ER tubules. *Cell* 147 1226–1231 22153070 10.1016/j.cell.2011.11.022 PMC3478066

[B32] Hu J. Shibata Y. Zhu P. P. Voss C. Rismanchi N. Prinz W. A. Rapoport T. A. Blackstone C. (2009). A class of dynamin-like GTPases involved in the generation of the tubular ER network. *Cell* 138 549–561 19665976 10.1016/j.cell.2009.05.025 PMC2746359

[B33] Kharroubi I. Ladriere L. Cardozo A. K. Dogusan Z. Cnop M. Eizirik D. L. (2004). Free fatty acids and cytokines induce pancreatic beta-cell apoptosis by different mechanisms: role of nuclear factor-kappaB and endoplasmic reticulum stress. *Endocrinology* 145 5087–5096 15297438 10.1210/en.2004-0478

[B34] Kim H. Kim A. Cunningham K. W. (2012). Vacuolar H^+^-ATPase (V-ATPase) promotes vacuolar membrane permeabilization and nonapoptotic death in stressed yeast. *J. Biol. Chem.* 287 19029–19039 22511765 10.1074/jbc.M112.363390 PMC3365936

[B35] Kucharz K. Wieloch T. Toresson H. (2011a). Potassium-induced structural changes of the endoplasmic reticulum in pyramidal neurons in murine organotypic hippocampal slices. *J. Neurosci. Res.* 89 1150–1159 21538461 10.1002/jnr.22646

[B36] Kucharz K. Wieloch T. Toresson H. (2011b). Rapid fragmentation of the endoplasmic reticulum in cortical neurons of the mouse brain in situ following cardiac arrest. *J. Cereb. Blood Flow Metab.* 31 1663–1667 21468089 10.1038/jcbfm.2011.37 PMC3170944

[B37] Lesage G. Bussey H. (2006). Cell wall assembly in *Saccharomyces cerevisiae.* *Microbiol. Mol. Biol. Rev*. 70 317–343 16760306 10.1128/MMBR.00038-05 PMC1489534

[B38] Levin D. E. (2011). Regulation of cell wall biogenesis in *Saccharomyces cerevisiae*: the cell wall integrity signaling pathway. *Genetics* 189 1145–1175 22174182 10.1534/genetics.111.128264 PMC3241422

[B39] Lisbona F. Rojas-Rivera D. Thielen P. Zamorano S. Todd D. Martinon F. Glavic A. Kress C. Lin J. H. Walter P. Reed J. C. Glimcher L. H. Hetz C. (2009). BAX inhibitor-1 is a negative regulator of the ER stress sensor IRE1alpha. *Mol. Cell* 33 679–691 19328063 10.1016/j.molcel.2009.02.017 PMC2818874

[B40] Lyman S. K. Schekman R. (1996). Polypeptide translocation machinery of the yeast endoplasmic reticulum. *Experientia* 52 1042–1049 8988244 10.1007/BF01952100

[B41] Madeo F. Carmona-Gutierrez D. Ring J. Buttner S. Eisenberg T. Kroemer G. (2009). Caspase-dependent and caspase-independent cell death pathways in yeast. *Biochem. Biophys. Res. Commun.* 382 227–231 19250922 10.1016/j.bbrc.2009.02.117

[B42] Madeo F. Herker E. Maldener C. Wissing S. Lachelt S. Herlan M. Fehr M. Lauber K. Sigrist S. J. Wesselborg S. Frohlich K. U. (2002). A caspase-related protease regulates apoptosis in yeast. *Mol. Cell* 9 911–917 11983181 10.1016/s1097-2765(02)00501-4

[B43] Oakes S. A. Opferman J. T. Pozzan T. Korsmeyer S. J. Scorrano L. (2003). Regulation of endoplasmic reticulum Ca^2+^ dynamics by proapoptotic BCL-2 family members. *Biochem. Pharmacol*. 66 1335–1340 14555206 10.1016/s0006-2952(03)00482-9

[B44] Orso G. Pendin D. Liu S. Tosetto J. Moss T. J. Faust J. E. Micaroni M. Egorova A. Martinuzzi A. McNew J. A. Daga A. (2009). Homotypic fusion of ER membranes requires the dynamin-like GTPase atlastin. *Nature* 460 978–983 19633650 10.1038/nature08280

[B45] Parmar V. M. Schroder M. (2012). Sensing endoplasmic reticulum stress. *Adv. Exp. Med. Biol.* 738 153–168 22399379 10.1007/978-1-4614-1680-7_10

[B46] Pineau L. Colas J. Dupont S. Beney L. Fleurat-Lessard P. Berjeaud J. M. Berges T. Ferreira T. (2009). Lipid-induced ER stress: synergistic effects of sterols and saturated fatty acids. *Traffic* 10 673–690 19302420 10.1111/j.1600-0854.2009.00903.x

[B47] Rizzuto R. Pinton P. Carrington W. Fay F. S. Fogarty K. E. Lifshitz L. M. Tuft R. A. Pozzan T. (1998). Close contacts with the endoplasmic reticulum as determinants of mitochondrial Ca^2+^ responses. *Science* 280 1763–1766 9624056 10.1126/science.280.5370.1763

[B48] Ron D. Walter P. (2007). Signal integration in the endoplasmic reticulum unfolded protein response. *Nat. Rev. Mol. Cell Biol.* 8 519–529 17565364 10.1038/nrm2199

[B49] Sano R. Hou Y. C. Hedvat M. Correa R. G. Shu C. W. Krajewska M. Diaz P. W. Tamble C. M. Quarato G. Gottlieb R. A. Yamaguchi M. Nizet V. Dahl R. Thomas D. D. Tait S. W. Green D. R. Fisher P. B. Matsuzawa S. Reed J. C. (2012). Endoplasmic reticulum protein BI-1 regulates Ca^2+^-mediated bioenergetics to promote autophagy. *Genes Dev.* 26 1041–1054 22588718 10.1101/gad.184325.111 PMC3360560

[B50] Schuck S. Prinz W. A. Thorn K. S. Voss C. Walter P. (2009). Membrane expansion alleviates endoplasmic reticulum stress independently of the unfolded protein response. *J. Cell Biol*. 187 525–536 19948500 10.1083/jcb.200907074 PMC2779237

[B51] Scorrano L. Oakes S. A. Opferman J. T. Cheng E. H. Sorcinelli M. D. Pozzan T. Korsmeyer S. J. (2003). BAX and BAK regulation of endoplasmic reticulum Ca^2+^: a control point for apoptosis. *Science* 300 135–139 12624178 10.1126/science.1081208

[B52] Shibata Y. Hu J. Kozlov M. M. Rapoport T. A. (2009). Mechanisms shaping the membranes of cellular organelles. *Annu. Rev. Cell Dev. Biol*. 25 329–354 19575675 10.1146/annurev.cellbio.042308.113324

[B53] Shibata Y. Shemesh T. Prinz W. A. Palazzo A. F. Kozlov M. M. Rapoport T. A. (2010). Mechanisms determining the morphology of the peripheral ER. *Cell* 143 774–788 21111237 10.1016/j.cell.2010.11.007 PMC3008339

[B54] Sidrauski C. Walter P. (1997). The transmembrane kinase Ire1p is a site-specific endonuclease that initiates mRNA splicing in the unfolded protein response. *Cell* 90 1031–1039 9323131 10.1016/s0092-8674(00)80369-4

[B55] Sorin A. Rosas G. Rao R. (1997). PMR1, a Ca2+-ATPase in yeast Golgi, has properties distinct from sarco/endoplasmic reticulum and plasma membrane calcium pumps. *J. Biol. Chem.* 272 9895–9901 9092527 10.1074/jbc.272.15.9895

[B56] Strayle J. Pozzan T. Rudolph H. K. (1999). Steady-state free Ca^2+^ in the yeast endoplasmic reticulum reaches only 10 microM and is mainly controlled by the secretory pathway pump pmr1. *EMBO J.* 18 4733–4743 10469652 10.1093/emboj/18.17.4733 PMC1171546

[B57] Tabas I. Ron D. (2011). Integrating the mechanisms of apoptosis induced by endoplasmic reticulum stress. *Nat. Cell Biol*. 13 184–190 21364565 10.1038/ncb0311-184 PMC3107571

[B58] Trusina A. Papa F. R. Tang C. (2008). Rationalizing translation attenuation in the network architecture of the unfolded protein response. *Proc. Natl. Acad. Sci. U.S.A.* 105 20280–20285 19075238 10.1073/pnas.0803476105 PMC2603256

[B59] Urano F. Wang X. Bertolotti A. Zhang Y. Chung P. Harding H. P. Ron D. (2000). Coupling of stress in the ER to activation of JNK protein kinases by transmembrane protein kinase IRE1. *Science* 287 664–666 10650002 10.1126/science.287.5453.664

[B60] Vashist S. Frank C. G. Jakob C. A. Ng D. T. (2002). Two distinctly localized p-type ATPases collaborate to maintain organelle homeostasis required for glycoprotein processing and quality control. *Mol. Biol. Cell* 13 3955–3966 12429838 10.1091/mbc.02-06-0090 PMC133606

[B61] Voeltz G. K. Prinz W. A. Shibata Y. Rist J. M. Rapoport T. A. (2006). A class of membrane proteins shaping the tubular endoplasmic reticulum. *Cell* 124 573–586 16469703 10.1016/j.cell.2005.11.047

[B62] Walter P. Ron D. (2011). The unfolded protein response: from stress pathway to homeostatic regulation. *Science* 334 1081–1086 22116877 10.1126/science.1209038

[B63] Wang X. Z. Harding H. P. Zhang Y. Jolicoeur E. M. Kuroda M. Ron D. (1998). Cloning of mammalian Ire1 reveals diversity in the ER stress responses. *EMBO J.* 17 5708–5717 9755171 10.1093/emboj/17.19.5708 PMC1170899

[B64] Watanabe R. Riezman H. (2004). Differential ER exit in yeast and mammalian cells. *Curr. Opin. Cell Biol.* 16 350–355 15261666 10.1016/j.ceb.2004.06.010

[B65] Watson P. Stephens D. J. (2005). ER-to-Golgi transport: form and formation of vesicular and tubular carriers. *Biochim. Biophys. Acta* 1744 304–315 15979504 10.1016/j.bbamcr.2005.03.003

[B66] West M. Zurek N. Hoenger A. Voeltz G. K. (2011). A 3D analysis of yeast ER structure reveals how ER domains are organized by membrane curvature. *J. Cell Biol.* 193 333–346 21502358 10.1083/jcb.201011039 PMC3080256

[B67] Wissing S. Ludovico P. Herker E. Buttner S. Engelhardt S. M. Decker T. Link A. Proksch A. Rodrigues F. Corte-Real M. Frohlich K. U. Manns J. Cande C. Sigrist S. J. Kroemer G. Madeo F. (2004). An AIF orthologue regulates apoptosis in yeast. *J. Cell Biol.* 166 969–974 15381687 10.1083/jcb.200404138 PMC2172025

[B68] Woehlbier U. Hetz C. (2011). Modulating stress responses by the UPRosome: a matter of life and death. *Trends Biochem. Sci*. 36 329–337 21482118 10.1016/j.tibs.2011.03.001

[B69] Wysocki R. Kron S. J. (2004). Yeast cell death during DNA damage arrest is independent of caspase or reactive oxygen species. *J. Cell Biol*. 166 311–316 15289493 10.1083/jcb.200405016 PMC2172262

[B70] Zimmermann R. Eyrisch S. Ahmad M. Helms V. (2011). Protein translocation across the ER membrane. *Biochim. Biophys. Acta* 1808 912–924 20599535 10.1016/j.bbamem.2010.06.015

[B71] Zong W. X. Li C. Hatzivassiliou G. Lindsten T. Yu Q. C. Yuan J. Thompson C. B. (2003). Bax and Bak can localize to the endoplasmic reticulum to initiate apoptosis. *J. Cell Biol.* 162 59–69 12847083 10.1083/jcb.200302084 PMC2172724

